# Remediable Anchylosis

**Published:** 1888-08

**Authors:** George H. Clark

**Affiliations:** Germantown, Philada.


### REMEDIABLE ANCHYLOSIS.

George H. Clark, Germantown, Philada.

(Read before the International Hahnemannian Association, at Niagara Falls, June, 1888.)

If an apology be necessary for offering facts with which every practical physician should be acquainted, the following case is tendered:

Some time ago, stopping in to see a gentleman on business, I found him with his right arm in a sling, and saw that he was unable to get his elbow away from the side, owing to anchylosis of the shoulder joint. Upon inquiry he informed me that he had a fall while walking about eight months before I saw him, striking the shoulder against a door-step. Since that time he had been under the care of his family physician, an allopath, who had used liniments without stint, and still he had a useless arm. I at once recognized a condition that is easily remedied—if one only knows how.

The function of a joint is movement. Nature provides a lubricant for this in the synovial fluid, which is being constantly poured out to keep the joint in working condition.

If, from any cause, the joint is kept at rest, the synovia will continue to be poured out, but as it is not being used, it will form about the joint, from its glutinous nature, a state of affairs similar to the provisional callus poured about a fracture. The longer the joint is kept at rest, the firmer becomes the hardened fluid, until, finally, it is almost as rigid as bone.

In the case above noted I saw this was the condition, and by firmly grasping the arm I was enabled to break up this hardened synovia by active movements of the shoulder joint, and in less than five minutes the gentleman was able to put on his coat and use his arm for other purposes, and in less than one week his arm was as useful as ever. Such is what a knowledge of facts enables one to accomplish.

In more chronic cases, before attempting to break up the adhesions, it is better to have the joint enveloped in flaxseed poultices for about one week, and rubbed several times daily with neat's-foot oil.

This will serve to soften the adhesions, and render their breaking up easier.

This method may be applied not only to joints that have received injury, but also to those which have become anchylosed from disease, the disease having disappeared.

In joints in which disease exists, it is not well to attempt to bring about movement by any harsh measures. The indications for thus manipulating joints are: Where there is ability to make some movement, but which is checked by pain, and a spot tender on pressure.

These tender spots are usually found in false anchylosis, and in manipulating the joint it is well to find this spot, and exert pressure upon it in attempting to make movements.

"The tender spot is usually found about the inner aspect of the joint. In the hip it is over the head of the femur, and about the centre of the groin; in the knee, at the lower edge of the internal condyle; in the elbow, over the internal condyle of the humerus; at the wrist, over the scaphoid or semi-lunar bone.

"The painful spot being discovered, the limb must be steadied on the proximal, and grasped on the distal side of the affected joint, and thumb pressure applied to the seat of the pain, and the joint sharply flexed, or flexed and extended, sometimes also abducted and adducted, as the case may demand. The direction of movement must depend mainly upen the direction of resistance."

From a little work entitled *On Bone Setting*, published by Macmillan & Co. some years ago, much that is of value on this subject may be found, and I advise every one to procure a copy of it, if possible.

To this work I hereby acknowledge my indebtedness.

